# Development of Gabapentin Expandable Gastroretentive Controlled Drug Delivery System

**DOI:** 10.1038/s41598-019-48260-8

**Published:** 2019-08-12

**Authors:** Issam B. Rimawi, Ramzi H. Muqedi, Feras I. Kanaze

**Affiliations:** 1 Faculty of Pharmacy, Nursing and Health Professions, Birzeit University, Al-Sahel Street 627, Ramallah, Palestine; 2 Samih Darwazah Institute for Pharmaceutical Industries, Faculty of Pharmacy, Nursing and Health Professions, Birzeit University, Al-Sahel Street 627, Ramallah, Palestine

**Keywords:** Epilepsy, Drug development

## Abstract

Expandable drug delivery systems are one of many gastroretentive delivery systems which have emerged during the last few years. Expandable systems are usually folded in a capsule and expand to dimensions greater than the pyloric sphincter upon contact with gastric fluid. This prevents them from being evacuated by gastric emptying. The main objective of developing such systems is to increase the residence time of a specific drug in stomach; controlling its release, increasing its bioavailability and decreasing its side effects and dosing frequency. An expandable gastroretentive drug delivery system containing Gabapentin was developed using experimental design (D-optimal reduced quadratic design). This system was able to unfold at stomach pH in less than 15 minutes and obtain a controlled release of 78.1 ± 4.7% in 6 hours following zero-order release kinetic model. It is rigid in stomach and its rigidity decreases at intestinal pH. FTIR analysis indicated the occurrence of hydrogen bonding in Gabapentin when present in the developed system, which might be responsible for the drug’s controlled release. XRD analysis indicated that Gabapentin physical properties changed from crystalline in the typical state to amorphous in the developed system.

## Introduction

**Figure 1:**
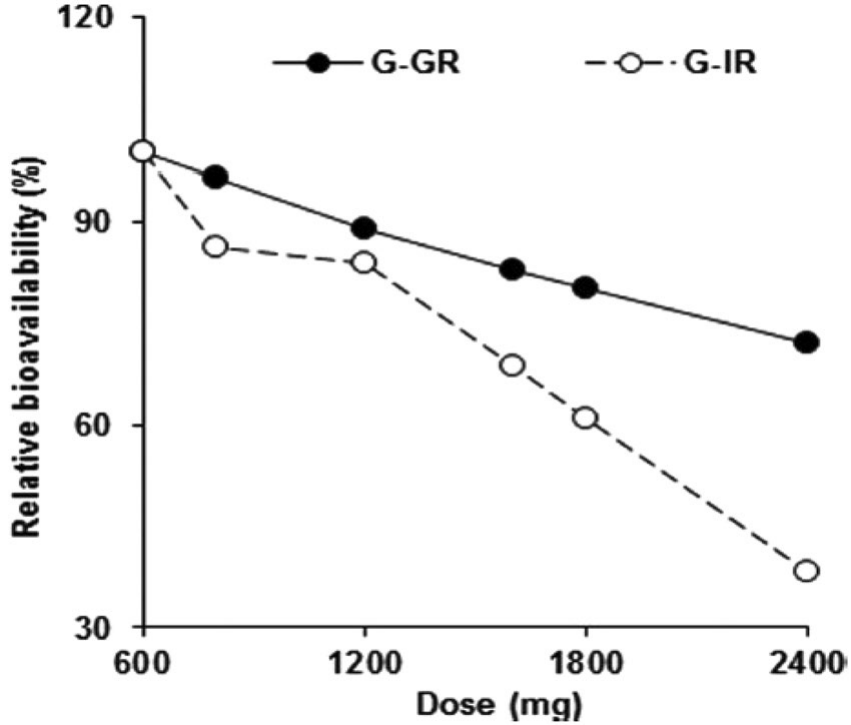
Relative bioavailability of swellable floatable gastroretentive Gabapentin (G-GR) vs immediate release Gabapentin (G-IR)^21^.

Gastroretentive drug delivery systems are intended to remain in stomach for prolonged periods. They include floating^1–3^, bioadhesive^4,5^, high density^6,7^, magnetic^8,9^ and expandable systems^10,11^. The diversity in these systems is owed to the numerous benefits obtained from designing them. These benefits include increased drug bioavailability, decreased side effects and dosing frequency, in addition to increased patient compliance^12^. Expandable systems expand, after being folded in capsule, once in contact with gastric fluid. This expansion should provide a system with dimensions greater than the pyloric sphincter in its relaxed state (12.8 ± 7 mm), which ensures mechanical resistance to evacuation^13,14^. The stomach contains an acidic medium which range from pH 1.1–4. Fed state increases the pH^15^, while fasted state lowers it^16–18^. It possesses an evacuating mechanism called gastric emptying, during which a series of contractions result in evacuating the stomach contents to the intestine through the pyloric sphincter^19^. This process occurs faster in fasted state compared to fed state. Studies have demonstrated that drugs taken on an empty stomach are usually evacuated within one hour from ingestion^19^. The two main factors affecting gastric retention of drug dosage forms are the fed or fasted state, and the size of the delivery system. A system which has dimensions greater than the pyloric sphincter in its relaxed state will have prolonged gastric residence time irrelevant to the fed or fated state. Gastroretentive delivery systems are mainly intended for drugs having a narrow absorption window, a biological half-life ranging from 2–8 hours and drugs taken in multiple daily doses^12,20^. Gabapentin has a narrow absorption window, an approximate half-life of 6 hours and is usually taken in multiple daily doses. As a result, it was considered appropriate for the developed gastroretentive system^21,22^. Gabapentin is mainly used as an anticonvulsant agent and for neuropathic pain^23^. Usually, the administered dose for epilepsy ranges from 0.9–3.6 g daily and could reach up to 1.8 g daily for neuropathic pain^23^. 4.8 g daily dose was reported to be well tolerated^23^. According to the biopharmaceutical classification system (BCS), Gabapentin is considered a class III drug. Its solubility is independent on the pH^21^. Its absorption mainly occurs in the jejunum and the duodenum^24–26^. L-amino acid transporters (LAT) are the main transporters responsible for the uptake of Gabapentin in the small intestine. Expression of LAT is decreased along the small intestine and is absent in the colon^27^. Saturation of these transporters prevents proportional increase in bioavailability with dose and usually occurs in immediate release dosage forms (Fig. 1)^21,25,27,28^. The developed expandable system represents a matrix system in the form of a layer. This system was developed using methodical optimization techniques based on experimental design (D-optimal reduced quadratic design). It is comprised of the active ingredient (Gabapentin), a plasticizer to increase the flexibility of the layer (Poloxamer P407), a mixture of hydrophobic polymers stable at stomach pH to control the drug release (Eudragit L100, S100, L100-55) and a swellable hydrophilic polymer which will expand as a result of fluid absorption (Gelatin). In the present research; drug release, unfolding, degradability and elasticity tests were performed on the developed formulations. Physical and chemical characteristics of Gabapentin were inspected using XRD and FTIR analyses, respectively.

## Results

### Antiadhesive excipients selection for developed layers unfolding test

The following antiadhesive excipients were intended to prevent developed layers stickiness and assist in the unfolding process (Table 1). A successful unfolding test is obtained when the tested layers unfold to at least 20 mm in 15 minutes. This test was performed using USP dissolution Apparatus II method and HCl medium (pH 1.2). Tested layers were removed from the dissolution apparatus after 10 and 15 minutes to measure their lengths.

**Table 1:** Different antiadhesive excipients effects on the total time required for the unfolding process (pH 1.2).

Antiadhesive excipient	Length of the tested layer(mm)	
	After 10 minutes	After 15 minutes
Talc	12	12
Microcrystalline cellulose	—*	—
Starch	10	12
Magnesium stearate	—	—
Magnesium stearate and talc	—	—
Magnesium stearate, citric acid and sodium bicarbonate	—	—
Talc, citric acid and sodium bicarbonate	18	20
Citric acid and sodium bicarbonate	21	24

*No unfolding occurred.

Citric acid and sodium bicarbonate were tested on three additional samples and provided positive results (i.e. length >20 mm). Consequently, this combination was selected as the ideal antiadhesive excipient.

### Drug release, capsule disintegration, unfolding (pH 1.2) and Young’s modulus tests

Drug release, capsule disintegration, unfolding (pH 1.2) and Young’s modulus tests results of the developed formulations are shown in Table 2.

**Table 2:** Drug release, capsule disintegration, unfolding (pH 1.2) and Young’s modulus tests results.

Formula	Cumulative drug release % (6 hours)	Capsule disintegration time (minutes)	Unfolding test	Young’s modulus (N/mm^2^)
F.A	81.8	2.1	Fail	0.0117
F.B	73.1	2.5	Fail	0.0079
F.C	49.8	3.6	N/A^*a^	N/A^*a^
F.D	60.0	2.4	N/A^*a^	N/A^*a^
F.E	91.2	3	Pass^*b^	0.0142
F.F	78.2	3.4	Pass	0.0067
F.G	77.5	2.8	Pass	Fail^*c^
F.H	84.9	3.7	Fail	0.0174
F.I	78.7	3.6	Fail	0.0086
F.J	89.5	4	Pass	0.0258
F.K	79.4	2.7	Fail	0.0115
F.L	82.8	3.6	N/A^*a^	N/A^*a^
F.M	91.3	2.4	Pass	0.0133
F.N	72.0	3.8	N/A^*a^	N/A^*a^
F.O	81.4	3.4	N/A^*a^	N/A^*a^
F.P	94.5	3	Fail	0.0164
F.Q	100	3.7	Pass	0.0258
F.R	94.6	2.8	N/A^*a^	N/A^*a^
F.S	98.6	2	N/A^*a^	N/A^*a^

^*a^Test is not applicable due to low layer elasticity, ^*b^A layer passes the unfolding test if it unfolds within 15 minutes of being in contact with the release test medium, ^*c^Failing in Young’s modulus test when the layer is cut upon stress.

**Figure 2:**
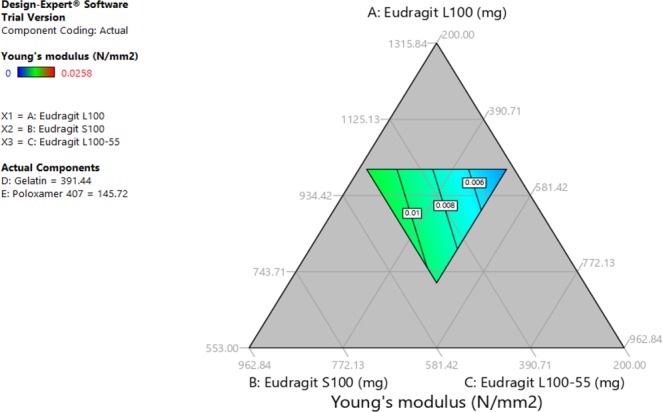
Excipients quantities relationship with Young’s modulus test.

**Figure 3:**
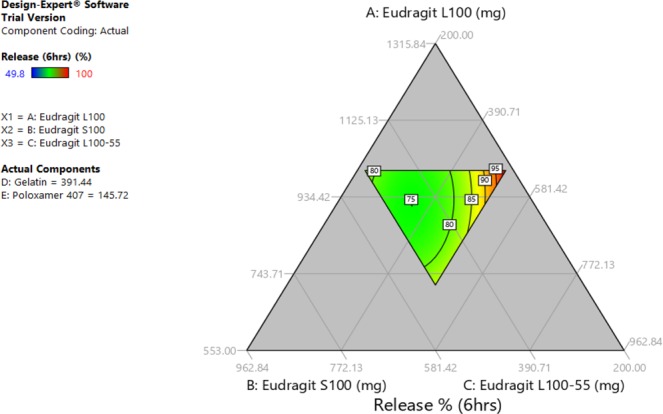
Excipients quantities relationship with drug release at 6 hours.

**Figure 4:**
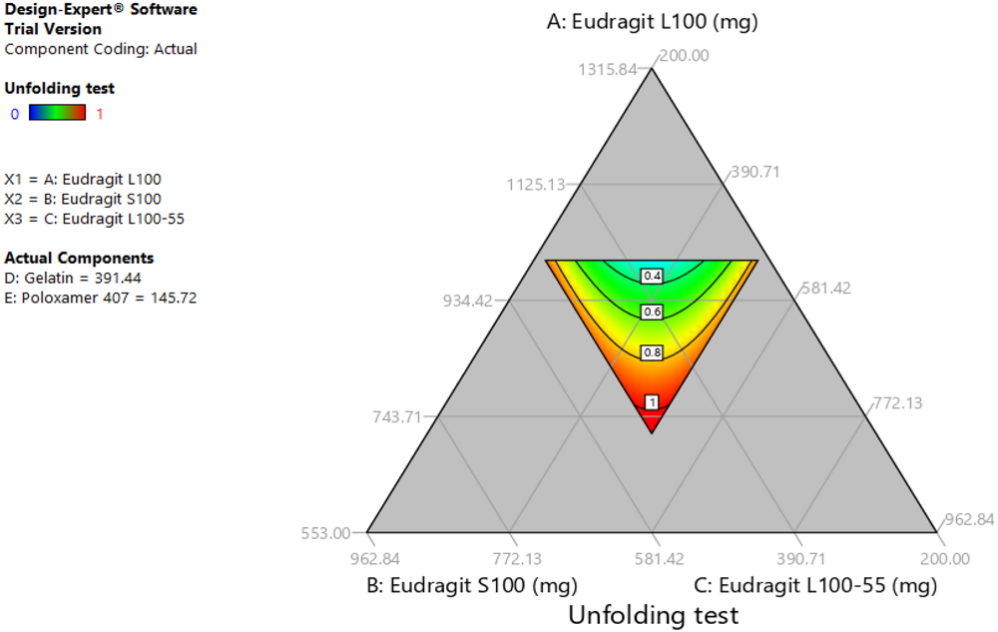
Excipients quantities relationship with the unfolding test.

**Figure 5:**
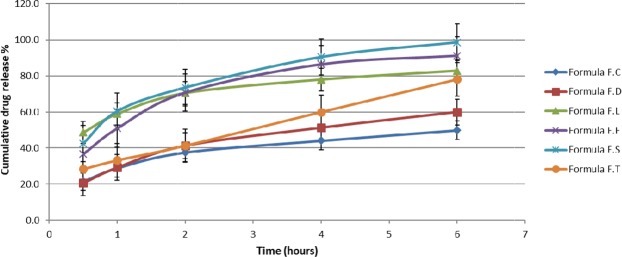
Drug release comparison between optimized formula F.T and different developed formulations in HCl medium. (n = 3, R.S.D < 5.6%).

Tests results data were entered to Design Expert software which provided ternary graphs that explain the relationship between drug release, unfolding and Young’s modulus tests with excipients quantities (Figs 2, 3 and 4). The three axis variables are Eudragit polymers. Gelatin and poloxamer P407 factor values were set to the centroid. Design Expert software also provided the optimized formula F.T (Table 3) after entering the suitable criteria (drug release of not less than 70% at 6 hours, a successful unfolding test and a Young’s modulus of not less than 0.015 N/mm^2^). Drug release, capsule disintegration, unfolding (pH 1.2) and Young’s modulus tests results of optimized formula are shown in Table 4. The optimized formula followed zero-order release kinetic model, which was not the case in previously developed formulations (Fig. 5, Table 5).

**Table 3:** Ingredients quantities in optimized formula F.T.

Formula	Ingredients (mg)					
	Gabapentin	Eudragit L100	Eudragit S100	Eudragit L100-55	Gelatin	Poloxamer P407
F.T	1591	1000	500	467	170	115

**Table 4:** Drug release, capsule disintegration, unfolding (pH 1.2) and Young’s modulus tests resultsof optimized formula F.T.

Formula	Cumulative drug release % (6 hours)	Capsule disintegration time (minutes)	Unfolding test	Young’s modulus (N/mm^2^)
F.T	*78.1 ± 4.8	2.4	Pass	0.017 ± 0.003

*n = 3 (formula F.T was manufactured 3 times).

**Table 5:** Release kinetic equation for optimal formula F.T and different developed formulations.

Release kinetic equation	Formula
Y = 4.7632x + 23.493, R² = 0.9085	F.C
Y = 6.7519x + 22.278, R² = 0.9335	F.D
Y = 5.6783x + 52.389, R² = 0.8587	F.L
Y = 9.4522x + 41.649, R² = 0.8611	F.E
Y = 9.4032x + 47.682, R² = 0.8949	F.S
Y = 9.0442x + 23.761, R² = 0.9997	F.T

### Assay test

The resultant assay test value following the mentioned dissolving method was 98 ± 1.2%. Gabapentin was dissolved in release test medium (HCl) and had an assay of 98.2 ± 0.9% after one month of storage at room temperature.

### Drug release and unfolding tests in acetate buffer medium (pH 4.1)

**Figure 6:**
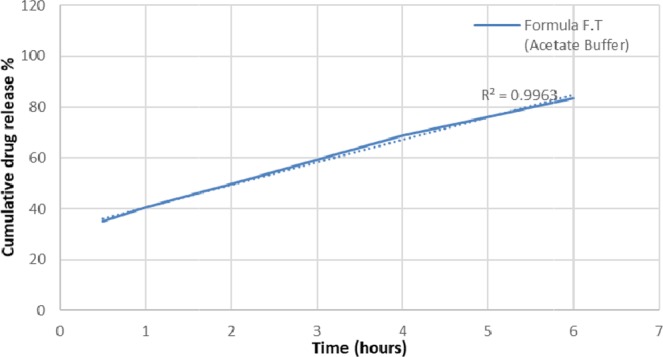
Mean drug release of 3 formulations of optimal formula F.T. in acetate buffer medium. (R.S.D < 5%).

Drug release and unfolding tests were performed on the optimal formula F.T (n = 3). 83.4 ± 5.3% release at 6 hours in acetate buffer (Fig. 6) and a successful unfolding test results were obtained.

### Degradability test (pH 6.5)

Developed layers demonstrate a significant increase in rigidity and thickness in wet state compared to dry state. Samples from optimized formula F.T were tested at pH 1.2 and 6.5 (USP Apparatus II). Young’s modulus for the wet samples tested at pH 6.5 was 0.110 N/mm^2^ after 5 hours, while for samples tested at pH 1.2 was 0.302 N/mm^2^ after 6 hours. Layers tested at pH 6.5 were 1.24 mm thick, while layers tested at pH 1.2 were 2.02 mm thick.

### Fourier-transform infrared spectroscopy (FTIR)

**Figure 7:**
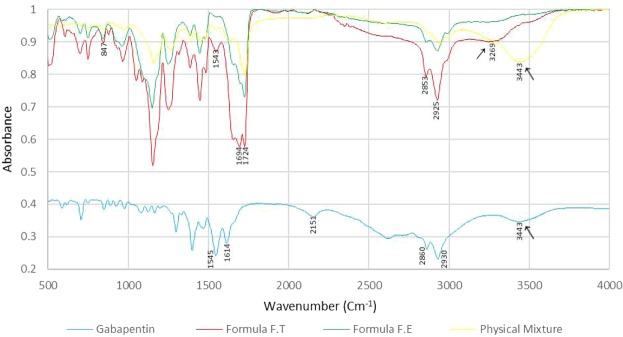
FTIR spectra for formulations F.T, F.E, pure Gabapentin and the physical mixture of Gabapentin with all excipients.

**Figure 8:**
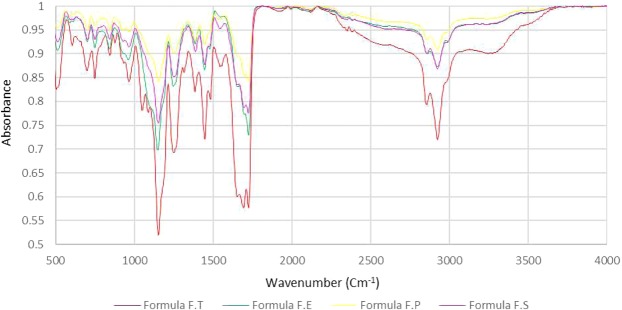
FTIR spectra for formulations F.E, F.P, F.S and F.T.

**Figure 9:**
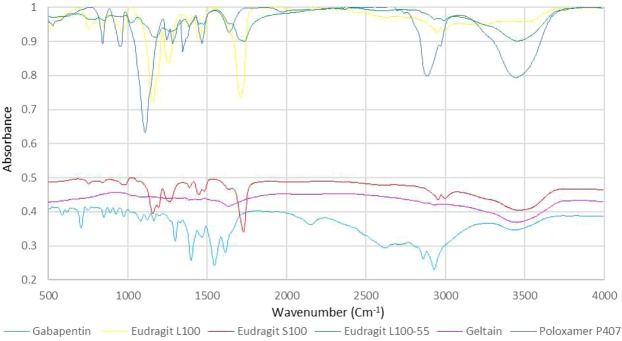
FTIR spectra for Gabapentin, gelatin, Eudragit L100, Eudragit S100, Eudragit L100-55 and poloxamer P407.

FTIR spectra for formulations F.T, F.E, pure Gabapentin and the physical mixture of Gabapentin with all excipients were obtained (Fig. 7). Regarding Gabapentin, it normally shows no peak in the –NH stretching regions (3500–3300 cm^−1^), since it is a zwitterion in the solid state^29,30^. The two peaks at 2930 cm^−1^ and 2860 cm^−1^ are stretching vibrations of –NH_3_^+^ ^31^. The peak at 2151 cm^−1^ represents stretching vibration of the side chain and/or CN group^32^. Peaks at 1545 cm^−1^ and at 1614 cm^−1^ are due to vibrations of NH_3_^+^ deformation and the ionized asymmetric carboxylate group, respectively^32^. The peaks at 3443 cm^−1^ in both Gabapentin and physical mixture are most probably due to hydroxyl groups stretching vibration of water molecules absorbed from moisture. Carboxylic acid hydroxyl group and CO stretching vibrations in the optimal formula F.T can be seen at 2925 cm^−1^ and 1724 cm^−1^, respectively. The shift in the hydroxyl group peak from 3443 cm^−1^ in Gabapentin and physical mixture to 3269 cm^−1^ in formula F.T indicates the occurrence of hydrogen bonding. This bonding is most probably related to the controlled release of the drug. Almost no peak can be observed in formula F.E in the hydroxyl region which indicates weaker hydrogen bonding. The absence of this peak may be associated with the relatively rapid release (i.e. relative to the optimized formula F.T) of the drug. Formulations F.S and F.P also displayed relatively rapid drug release and exhibited similar characteristics to formula F.E in FTIR (Fig. 8). The split in the carbonyl peak at 1694 cm^−1^ observed only in developed formulations is most probably an overtone of the 847 cm^−1^ original peak (Fermi resonance). FTIR spectra of pure Gabapentin and each excipient alone were also obtained (Fig. 9). Variations on the major chemical groups of Gabapentin before and after being involved in the developed formulations were studied. Main characteristic bands of Gabapentin were observed at 2930 cm^−1^, 2860 cm^−1^ (stretching vibrations of −NH_3_^+^) and at 1545 cm^−1^. The characteristic stretching bands observed in the optimal formula F.T at 2925 cm^−1^, 2853 cm^−1^ and at 1543 cm^−1^ indicate that no change has occurred on the major chemical groups of Gabapentin after being involved in the developed formulations and proves that it is compatible with the excipients used in theses formulations.

### X-Ray powder diffraction (XRD)

**Figure 10:**
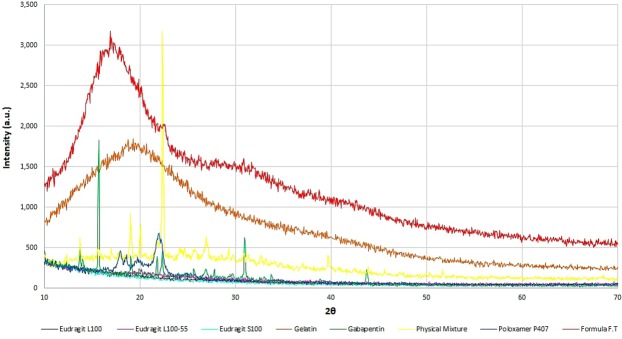
XRD patterns of pure Gabapentin, optimized formula F.T, the physical mixture of Gabapentin with all excipients and each excipient alone.

XRD patterns of pure Gabapentin, optimal formula F.T, the physical mixture of Gabapentin with all excipients and each excipient alone were obtained (Fig. 10). The major characteristics peaks of Gabapentin can be observed at 2θ = 15.7, 19.2, 22.9 and 31. These peaks could be also seen in the physical mixture of the ingredients. XRD patterns of pure Gabapentin revealed that it was in crystalline state before being involved in the developed formula. XRD patterns of the physical mixture also indicate a crystalline state. On the other hand, XRD diffraction patterns of the developed formula showed a broad peak which represents a typical profile of an amorphous material. These patterns assure that Gabapentin physical state was changed from crystalline to amorphous after being involved in the developed formula.

## Discussion

Regarding antiadhesive excipients used in the unfolding test, citric acid and sodium bicarbonate were grinded using mortar and pestle and spread as fine powder on the previously prepared layers. This combination produces CO_2_ gas which pushes the layer folded parts away from each other. It also decreases time required for capsule disintegration. The ideal ratio of citric acid to sodium bicarbonate was 1:10, respectively. An increase in citric acid ratio would lead to aggregates formation during grinding it with sodium bicarbonate which decreases the resultant powder stickiness to the prepared layers (i.e. sodium bicarbonate-citric acid grinded powder is more effective when it is trapped in between the developed layers folded parts as it helps push the folded parts away from each other). In the developed formula F.T, zero-order release kinetics was obtained, as a straight line with high correlation coefficient(R^2^) value resulted when plotting the cumulative drug release with time^33,34^. This was not the case in previously developed formulations (Table 5). Based on the release kinetic equation obtained (Y = 9.0442x + 23.761), 8.4 ± 0.62 hours are required to release 100% of the active ingredient. Comparedto the Accordion Pilldrug delivery system^11^; the developed system was able to control the drug release and expand at gastric pH using one layer instead of three. Gabapentin assay test results were within the acceptable limits andindicated that it is stable for one month of storage at room temperature after being dissolved in HCl. This result also assures that no degradation has occurred on Gabapentin while performing the drug release test in HCl medium for 6 hours. Drug release and unfolding tests resultsof the optimized formula F.T in acetate buffer medium (pH 4.1) which represents the higher pH of the stomach^15^ weresimilar to those obtained in HCl medium (pH 1.2) which represents the lower pH of the stomach^15^. These results prove that the change in gastric pH (as a result of food intake, disease, drugs, etc.) will not change the release or the expansion of the developed system. During the degradability test, both rigidity and thickness decreased significantly at intestinal pH (6.5) compared to rigidity and thickness results obtained at stomach pH (1.2). These results indicate that the developed system is more rapidly disintegrated and more elastic at intestinal pH and prove that the developed system will not remain in the intestines for prolonged periods, causing side effects, if premature evacuation occurs.

## Conclusion

The purpose of this study was to develop an expandable drug delivery system which extends Gabapentin release for at least 6 hours and retains in stomach for prolonged periods irrelevant to fed/fasted state. This extension usually involves enhanced bioavailability, deceased side effects and dosing frequency. During this study, one layered expandable gastroretentive controlled delivery system containing Gabapentin was developed using design of experiments. This system was able to unfold in less than 15 minutes, which ensured the avoidance of premature evacuation. Drug release followed zero-order release kinetic model and was successfully extended to at least 6 hours. About 8.4 hours are required to release all of the Gabapentin present in the system. Youngs’s modulus test result was above the set limit (0.015 N/mm^2^) and indicated high rigidity in stomach. Degradability test results demonstrated significant decrease in the system’s rigidity at intestinal pH. FTIR analysis proved that Gabapentin is compatible with the excipients used in the developed formulations and indicated the occurrence of hydrogen bonding in Gabapentin after being involved in the developed system. This bonding can be related to the controlled release of the drug. The shift in the physical state of Gabapentin from crystalline in typical state to amorphous in the developed system was confirmed by XRD analysis.

## Methodology

### HPLC apparatus

High performance liquid chromatography (HPLC) was used for assay and drug release studies of the prepared layers. HPLC analyses were performed using Agilent HPLC 1200 Seriesconsisting of a degasser (Model G1379B), a binary pump (Model G1312A), auto sampler ALS (Model G1329A), auto sampler thermostat FC/ALS Therm (Model G1330B), thermostat column compartment (Model G1316A) and a variable wavelength detector (Model G1314B). Separation was performed on Thermo Scientific Hypersil C18 column (150 * 4.6 mm, 5 µm BDS). The mobile phase consisted of buffer (1.2 g KH_2_PO_4_/1 liter water, adjusted to pH 6.9 with KOH)/Acetonitrile (90:10, v/v). The HPLC system was operated at a flow rate of 1.0 ml/min at 40 °C. The UV detector was set at 200 nm. Method used in HPLC analysis was according to the USP with some modifications. The analytical method was validated (results are not shown).

### System preparation using solvent evaporation method

**Figure 11:**
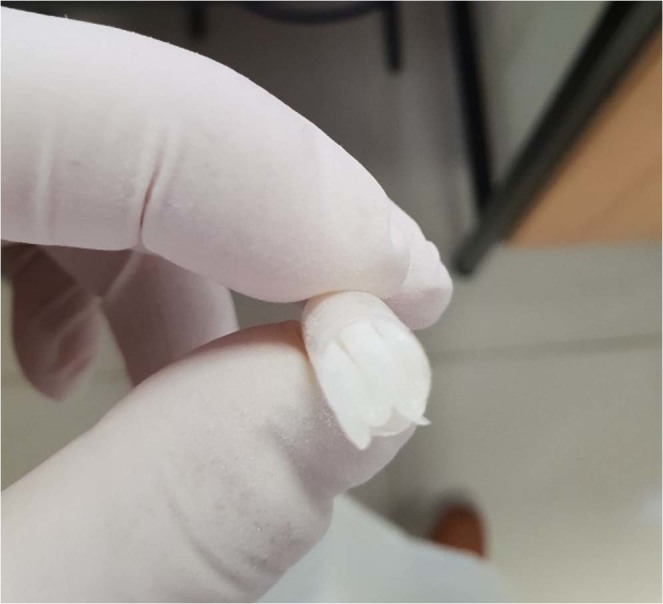
Prepared layer folded in a “00” sized hard gelatin capsule.

Several techniques were attempted to obtain a method which leads to a miscible mixture of the ingredients. Melting the ingredients together led to an immiscible mixture. Dissolving the ingredients in a common solvent, which can be easily removed, was found to be the most suitable method. Water, absolute ethanol and a mixture of both solvents were used to dissolve the ingredients. Absolute ethanol was the most suitable common solvent which led to a homogenous mixture. Several experiments were performed using different excipients (ex. HPMC, PEG400, Polyethylene glycol, KOH, PVP K-30) until the most suitable combination was selected based on performed tests results. 5 ml of absolute ethanol were added to a beaker and warmed using hot plate. Gradual addition of the ingredients with mixing was done starting with the hydrophobic polymers which were the hardest to dissolve. Eudragit L100-55 was added, followed by Eudragit L100 and S100. Additional 2.5 ml of the solvent were added followed by gelatin, Gabapentin and poloxamer P407. Mixing was done until a homogeneous mixture was obtained. The mixture was then poured in a glass mold and heated in the oven at 70 °C for 2 hours to ensure solvent evaporation and system solidification. The glass mold was covered with an antiadherent plastic bag originally used for cooking prior to mixture addition, in order to assist in dried layer removal. The resultant layer was stored in a desiccator for 2 days then cut to the required dimensions (20*30 mm^2^), weighed, folded manually into accordion shape and placed in a “00” sized hard gelatin capsule (Fig. 11). Developed layers weight and thickness, were about 450 mg and 1 mm, respectively.

### Experimental design

Several formulations were prepared using solvent evaporation method (Table 6). Each was tested for unfolding, elasticity and drug release. These formulations were provided by Design Expert software based on tests results obtained from previous scanning experiments to find the suitable concentration of each ingredient. D-optimal reduced quadratic design was used by the software.

**Table 6:** Ingredients quantities in developed formulations proposed using Design Expert software.

Formula	Ingredients (mg)					
	Gabapentin	Eudragit L100	Eudragit S100	Eudragit L100-55	Gelatin	Poloxamer P407
F.A	1591	703	500	350	500	0.200
F.B	1591	1000	500	326	226	0.200
F.C	1591	1000	500	500	253	—
F.D	1591	1000	500	253	500	—
F.E	1591	853	200	500	500	200
F.F	1591	901	370	370	500	110
F.G	1591	853	500	200	500	200
F.H	1591	1000	353	200	500	200
F.I	1591	553	500	500	500	200
F.J	1591	1000	500	200	426	126
F.K	1591	703	350	500	500	200
F.L	1591	1000	200	353	500	200
F.M	1591	753	500	500	300	200
F.N	1591	1000	253	500	500	—
F.O	1591	753	500	500	500	—
F.P	1591	753	500	500	300	200
F.Q	1591	1000	500	500	100	153
F.R	1591	1000	326	500	226	200
F.S	1591	1000	200	500	353	200

### Performed tests

#### Assay test

Numerous techniques were used to dissolve the developed layers and to obtain the highest assay test result. Different solvents were used (ex. DMSO, water, absolute ethanol, the mobile phase) to dissolve the tested layers. Sonication, shaking, and heating were tried to help extract the active ingredient from the tested layers. After several attempts, the optimal procedure was as follows. A part of each developed layer intended to be tested was cut into small pieces. These pieces were weighed and placed in a 50 ml volumetric flask containing the solvent absolute ethanol. The flask was then sonicated for 30 minutes. After which shaking was performed for 1 hour at room temperature. 5 ml was withdrawn from the resultant solution and diluted to 50 ml with the mobile phase. About 2 ml was withdrawn from the diluted solution and placed in a vial to be tested using HPLC. Solution stability test was performed on Gabapentin and was found to be stable for one month at room temperature.

#### Drug release test

**Figure 12:**
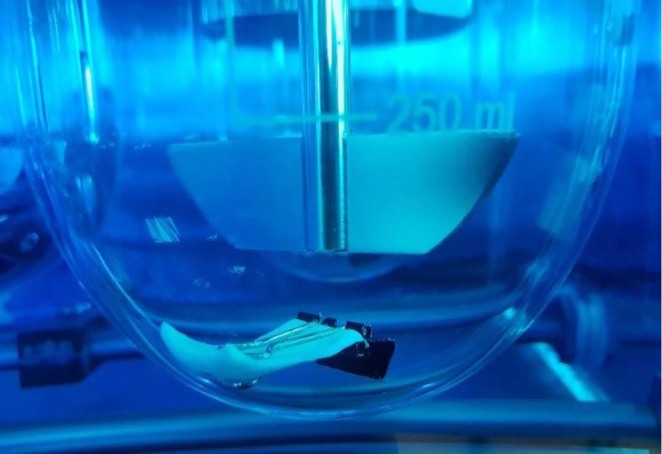
Drug release test performed on a layer held by paperclips.

Drug releasetest was performed using USP apparatus II method. Volume of medium was 500 ml. Sampling was performed at 0, 0.5, 1, 2, 4, 6 hours. Sample withdrawn was 2.5 ml. Sink conditions were maintained. Medium used in performed tests was hydrochloricacid medium (pH 1.2), which represents the lower pH of the stomach. An additional test using acetate buffer medium (pH 4.1) which represents the higher pH of the stomach was performed on the final optimized formula. Paperclips were used to hold the layers to the bottom of the bucket during layers development for the purpose of drug release studies (Fig. 12).

#### Unfolding test

**Figure 13:**
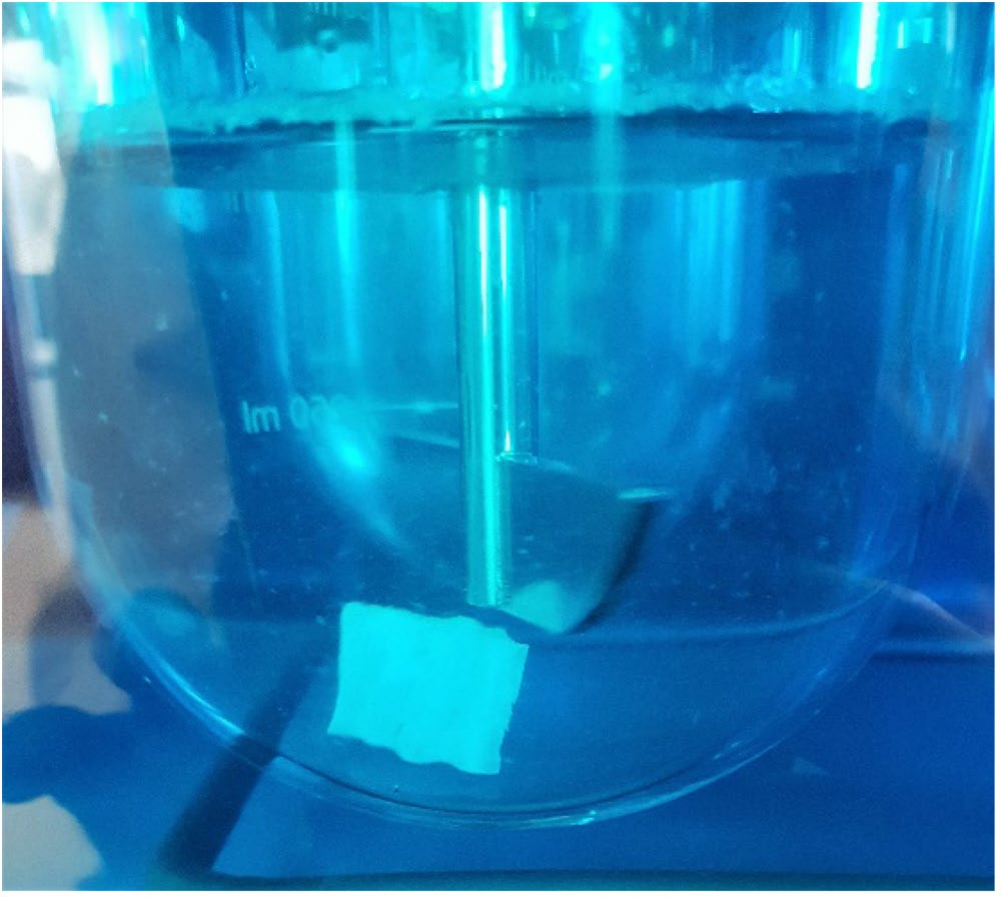
Unfolded layer observed after capsule disintegration.

Unfolding test was performed using USP Apparatus II method (Fig. 13). Hydrochloric acid medium (pH 1.2) was used to test all samples. An additional test using acetate buffer medium (pH 4.1) was performed on the final optimized formula. Capsules were disintegrated within 3–5 minutes. Developed layers should unfold within 15 minutes of ingestion to prevent premature evacuation. The unfolded layer dimensions should exceed the pyloric sphincter dimensions in its relaxed state (i.e. 20 mm). The layers displayed increased stickiness upon contact with the release test medium. Different antiadhesive excipients were used to prevent layer sticking.

#### Young’s modulus

**Figure 14:**
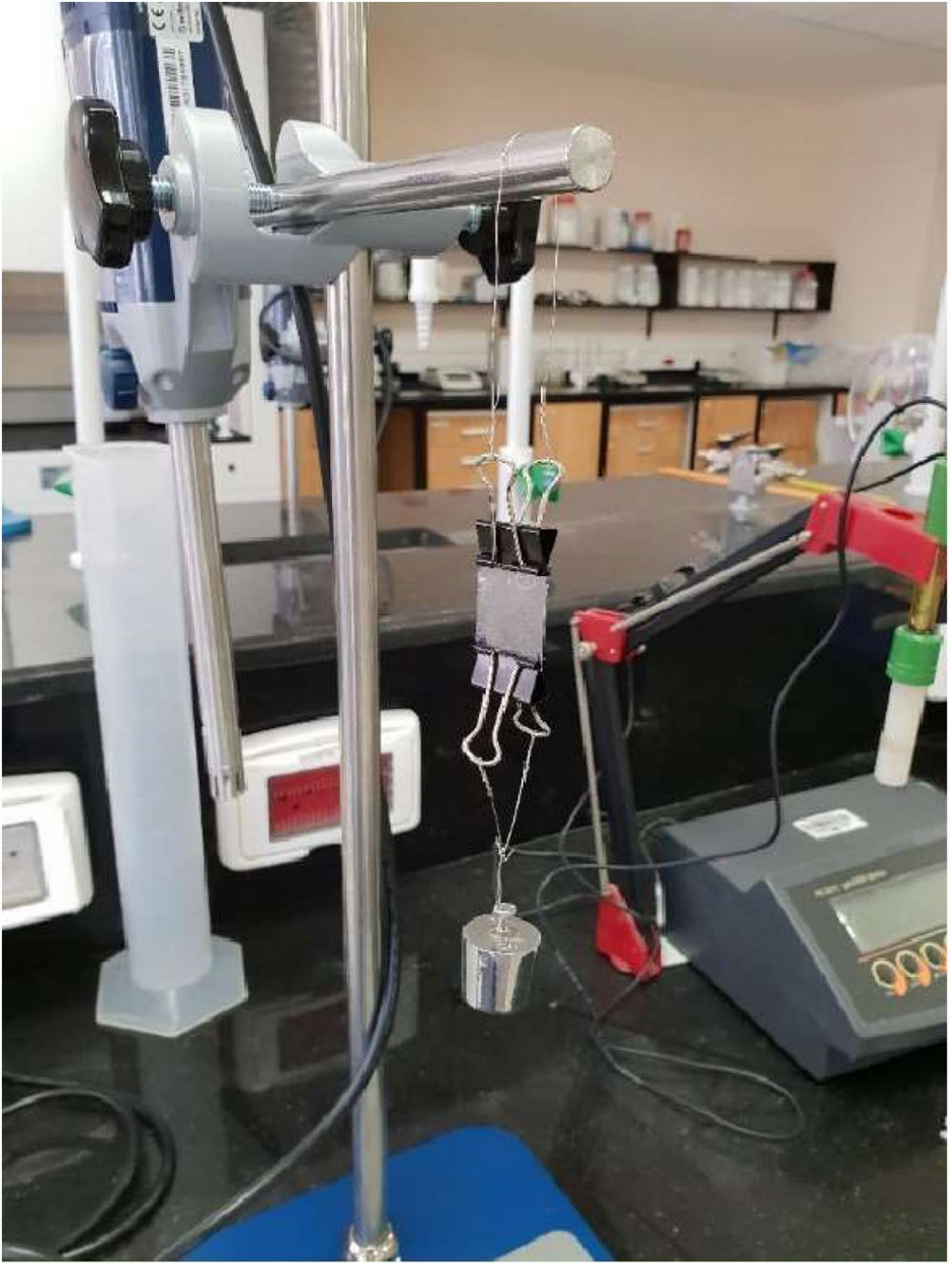
Young’s modulus test performed manually.

Young’s modulus or elasticity test is a measure of a material’s resistance to deformation upon stress. It is equal to stress over strain. Higher Young’s modulus value indicates higher layer rigidity. The most rigid layer will represent the optimal layer, as it will resist unplanned deformation (i.e. after unfolding) resulted from gastric emptying forces. The test was performed manually by attaching the layer to a retort stand from one side and to a weight from the other (Fig. 14). Same weight was used to test all samples. Time under stress was 2 minutes. Dimensions were measured using a digital caliper.

#### Degradability test

This test was performed to inspect the degradability of the optimized formula at intestinal pH using potassium phosphate buffer, pH 6.5. The layer was placed directly in the medium without being first loaded in a capsule. Its rigidity should decrease at alkaline pH. Rigidity was inspected manually using Young’s modulus test. Results will help us predict the layer’s behavior in the intestines in case of premature evacuation.

#### Fourier-transform infrared spectroscopy (FTIR)

FTIR analysis is a technique used to study and identify the interactions between reactive groups in different compounds. This analysis helps us understand the nature of the bonds which have formed during different formulations development and relate test results with the release obtained. FTIR analysis was performed on different developed formulations, the physical mixture of the ingredients used in theses formulations and on each ingredient alone. FTIR compatibility test was also performed to study the compatibility of Gabapentin with the excipients used in developed formulations. FTIR spectra were obtained test using Tensor II FTIR Spectrometer, Bruker. The spectra of raw materials were collected by compression of about 1% wt. in KBr tablets, while for developed layers the spectra were collected using small pieces of the layer without being first compressed in KBr tablets. The IR spectra were obtained at spectral region of 400–4000 cm^−1^ using 4 cm^−1^ resolution.

#### X-Ray powder diffraction (XRD)

XRD analysis is used to determine the physical properties of tested samples. It differentiates between crystalline and amorphous materials. Results will demonstrate the physical state of Gabapentin before and after being involved in the developed system. XRD analyses were performed on Miniflex 600x-ray diffraction unit, Rigaku according to the following conditions. 40 kV F.F tube, 15 mA beam, scintillation counter (Kβ filter) detector, slit conditions DS/SS = 1.25°, RS = 0.3 mm, incident side and receiving side Soller slit = 5°, incident height limiting slit = 10 mm, scan speed = 2°/min.
